# Systematic identification of yeast cell cycle transcription factors using multiple data sources

**DOI:** 10.1186/1471-2105-9-522

**Published:** 2008-12-05

**Authors:** Wei-Sheng Wu, Wen-Hsiung Li

**Affiliations:** 1 Department of Evolution and Ecology, University of Chicago, 1101 East 57th Street, Chicago, IL, 60637, USA; 2 Research Center for Biodiversity and Genomics Research Center, Academia Sinica, Taipei, Taiwan

## Abstract

**Background:**

Eukaryotic cell cycle is a complex process and is precisely regulated at many levels. Many genes specific to the cell cycle are regulated transcriptionally and are expressed just before they are needed. To understand the cell cycle process, it is important to identify the cell cycle transcription factors (TFs) that regulate the expression of cell cycle-regulated genes.

**Results:**

We developed a method to identify cell cycle TFs in yeast by integrating current ChIP-chip, mutant, transcription factor binding site (TFBS), and cell cycle gene expression data. We identified 17 cell cycle TFs, 12 of which are known cell cycle TFs, while the remaining five (Ash1, Rlm1, Ste12, Stp1, Tec1) are putative novel cell cycle TFs. For each cell cycle TF, we assigned specific cell cycle phases in which the TF functions and identified the time lag for the TF to exert regulatory effects on its target genes. We also identified 178 novel cell cycle-regulated genes, among which 59 have unknown functions, but they may now be annotated as cell cycle-regulated genes. Most of our predictions are supported by previous experimental or computational studies. Furthermore, a high confidence TF-gene regulatory matrix is derived as a byproduct of our method. Each TF-gene regulatory relationship in this matrix is supported by at least three data sources: gene expression, TFBS, and ChIP-chip or/and mutant data. We show that our method performs better than four existing methods for identifying yeast cell cycle TFs. Finally, an application of our method to different cell cycle gene expression datasets suggests that our method is robust.

**Conclusion:**

Our method is effective for identifying yeast cell cycle TFs and cell cycle-regulated genes. Many of our predictions are validated by the literature. Our study shows that integrating multiple data sources is a powerful approach to studying complex biological systems.

## Background

Eukaryotic cell cycle is a complex process and is precisely regulated at many levels. One important aspect of this regulation is at the transcriptional level. That is, many genes specific to the cell cycle are transcribed just before they are needed [1]. To have a good understanding of the cell cycle, it is essential to identify the cell cycle-regulated genes and their transcriptional regulators. DNA microarray analysis has revealed that the expression levels of ~800 genes vary in a periodic fashion during the yeast cell cycle, but little is known about the transcriptional regulation of most of these genes [2,3]. To fill this gap, we aim to identify the cell cycle transcription factors (TFs) that regulate the cell cycle-regulated genes inferred by DNA microarray analysis [2].

Two major approaches have been proposed to identify cell cycle TFs. First, clustering and motif-discovering algorithms have been applied to cell cycle gene expression data to find sets of co-expressed genes and plausible binding motifs of their TFs [2,4]. This approach has been expanded to incorporate existing knowledge about the genes, such as cellular functions [5] or promoter sequence motifs [6]. However, this approach provides only indirect evidence of genetic regulatory interactions and does not directly identify the relevant cell cycle TFs. Second, the ChIP-chip technique was developed to identify physical interactions between TFs and promoters. Using ChIP-chip data, Simon *et al*. [3] investigated how the yeast cell cycle gene expression program is regulated by the nine known major transcriptional activators. Later, Lee *et al*. [7] constructed a network of TF-promoter interactions and Harbison *et al*. [8] constructed an initial map of yeast's transcriptional regulatory code. However, ChIP-chip data alone cannot tell whether a TF is an activator or a repressor and, most importantly, ChIP-chip data are noisy and, depending on the chosen *p*-value cutoff, may include many false positive or false negative TF-promoter binding relationships. For example, if the *p*-value cutoff is chosen to be 0.001, a false negative rate of ~24% in determining TF-promoter binding was estimated [8].

**Figure 1:**
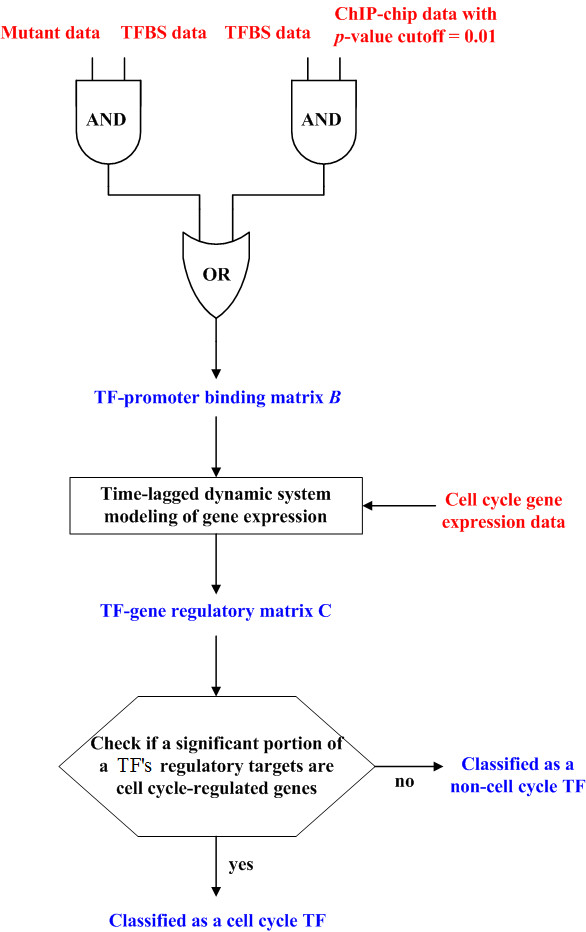
Flowchart of the procedure of our method.

To overcome the weakness of the above two approaches, we develop a method (details shown in Figure 1) to systematically identify cell cycle TFs by combining four data sources: transcription factor binding site (TFBS), mutant, ChIP-chip, and cell cycle gene expression data. In order to reduce the high false negative rate of the ChIP-chip data, we use current TFBS data [9,10] to avoid using a stringent *p*-value threshold (≤ 0.001) to determine TF-promoter binding. We assume that a TF binds to a specific promoter if (1) the *p*-value for the TF to bind the promoter is ≤ 0.01 in ChIP-chip data and (2) the promoter contains one or more binding sites of the TF. That is, we allow the *p*-value cutoff to be relaxed to 0.01 but the TF-promoter binding event must be supported by the TFBS data.

It is known that the ChIP-chip technique can only detect those TF-promoter binding events that happen in the same physiological condition in which the ChIP-chip experiment is conducted, so it can potentially miss many TF-promoter binding events. We use the mutant data [10] and the TFBS data [9,10] to rescue some of these false negative TF-promoter binding events. We assume that a TF binds to a specific promoter if (1) the disruption of the TF results in a significant change of expression of the gene that has the specific promoter and (2) the promoter contains one or more binding sites of the TF. That is, the TF-promoter binding event can be assumed without using ChIP-chip data if it is supported by both the mutant and the TFBS data. This step can rescue some plausible TF-promoter binding events that are missing in the current ChIP-chip data.

From the above procedure, we can derive a high-confidence TF-promoter binding matrix (see Methods). However, binding of a TF to the promoter of a gene does not necessarily imply regulation. A TF may bind to the promoter of a gene but has no regulatory effect on that gene's expression. Hence, additional information is required to solve this ambiguity inherent in the TF-promoter binding matrix. In this study, we use the additional information provided by the yeast cell cycle gene expression data [2] to solve this problem. We use a time-lagged dynamic system model of gene regulation to describe how the target gene's expression during cell cycle is controlled by the TFs that bind to its promoter (inferred from the TF-promoter binding matrix). Among these bound TFs, those that have significant regulatory effects on the target gene's expression can be extracted (see Methods). From this procedure, we can refine the TF-promoter binding matrix into a high-confidence TF-gene regulatory matrix. Each TF-gene regulatory relationship in this matrix is supported by at least three data sources: gene expression, TFBS, and ChIP-chip or/and mutant data. From the high-confidence TF-gene regulatory matrix, the regulatory targets of each of the 203 TFs in yeast can be inferred. Finally, a TF is said to be a cell cycle TF if a statistically significant portion of its regulatory targets are cell cycle-regulated genes.

## Results

### Identification of 17 cell cycle TFs

By integrating current ChIP-chip, mutant, TFBS, and yeast cell cycle gene expression data, our method identified 17 cell cycle TFs (Table 1). Among them, 12 are known cell cycle TFs according to the MIPS database [11], including the nine well-known major cell cycle TFs (Ace2, Fkh1, Fkh2, Mbp1, Mcm1, Ndd1, Swi4, Swi5, and Swi6), and Cin5, Cst6, and Stb1.

**Table 1:** The 17 identified cell cycle TFs

TF name	Hypergeometric *p*-value	MG_1_	G_1_	S	SG_2_	G_2_M
**Fkh2**	< 10^-11^		C [12,13]		E [3,7]	E [21,22]

**Mbp1**	< 10^-11^		E [3,19]	C [12,13]		

**Mcm1**	< 10^-11^	E [17,18]		C [12]		E [21,22]

**Swi4**	< 10^-11^	C [2,12,13,26]	E [3,19]	E [7,20]		C [12,13]

**Swi6**	< 10^-11^		E [3,19]		E [3,7]	

Tec1	1.8 × 10^-11^	C [2,12,13]	C [13]			C [12]

**Ndd1**	3.5 × 10^-11^		C [12,13]			E [21,22]

Ash1	9.6 × 10^-10^	C [2]	C [14]			

Ste12	2.2 × 10^-9^	C [12,13,26]	C [13,26]			N

**Swi5**	6.2 × 10^-9^	E [16]				

**Fkh1**	6.9 × 10^-9^				E [3,7]	E [21,22]

Rlm1	2.1 × 10^-7^	C [13]	C [13]	N		

**Stb1**	1.3 × 10^-6^		C [2,13,26]			

Stp1	4.7 × 10^-5^					N

**Ace2**	1.2 × 10^-4^	E [16]				

**Cin5**	1.8 × 10^-4^					C [13]

**Cst6**	3.5 × 10^-4^	N				N

The twelve known cell cycle TFs (according to the MIPS database) are bold-faced and colored blue. The 17 identified TFs are ordered by the confidence of being cell cycle TFs (according to the hypergeometric *p*-value calculated using Equation (4)). For each identified cell cycle TF, the cell cycle phases in which the TF functions are shown. "E" means that the prediction is supported by experimental evidence, "C" means that the prediction is supported by previous computational studies, and "N" stands for our novel prediction.

**Figure 2:**
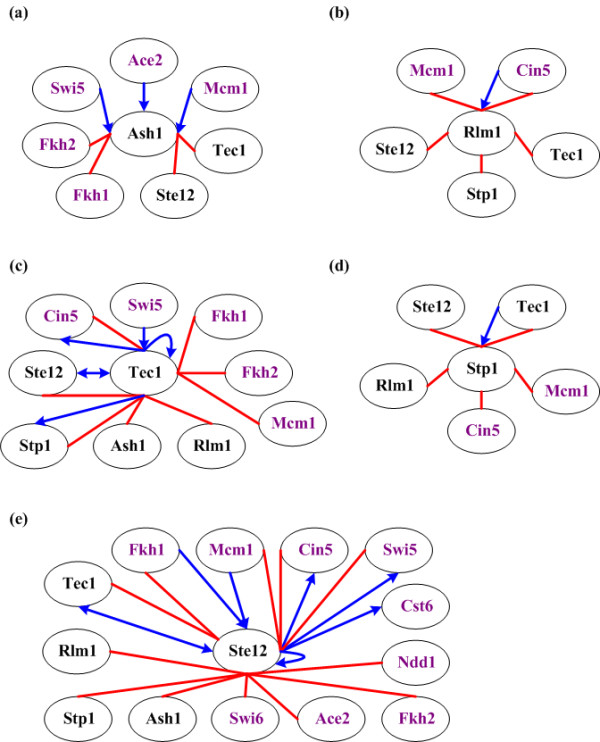
**Interactions between a novel cell cycle TF and the other identified cell cycle TFs**. The physical or genetic interactions between a novel cell cycle TF ((a) Ash1, (b) Rlm1, (c) Tec1, (d) Stp1, and (e) Ste12) and the other identified cell cycle TFs are shown. Each oval indicates an identified cell cycle TF. A TF name is colored purple if it is a known cell cycle TF but black otherwise. Two ovals are connected by an undirected red line if these two TFs have physical interactions indicated by the current protein-protein interaction data [48]. Two ovals are connected by a directed blue line if the two TFs have genetic interactions indicated by ChIP-chip or/and mutant data [10]. For example, Ace2→Ash1 means that either TF Ace2 binds to the promoter of gene *ASH1 *or the disruption of TF Ace2 results in a significant change of the expression of gene *ASH1*.

The remaining five predicted novel cell cycle TFs (Ash1, Rlm1, Ste12, Stp1 and Tec1) are supported by three lines of evidence. First, each novel cell cycle TF has physical or genetic interactions with some known cell cycle TFs (see Figure 2), suggesting that these five TFs may play a role in the yeast cell cycle. Second, four (Ash1, Rlm1, Ste12 and Tec1) of the five predicted novel cell cycle TFs have also been predicted in previous computational studies [12-14]. Third, Ash1, Rlm1, Stp1 and Tec1 were predicted to be cell cycle-regulated by previous studies [1,15]. Being cell cycle regulated themselves, these TFs may play a role in the cell cycle process.

### The cell cycle phases in which a cell cycle TF functions

After identifying the cell cycle TFs, it is desirable to determine in which cell cycle phases a cell cycle TF functions. We regard that a cell cycle TF functions in the *X *phase (*X *= MG_1_, G_1_, S, SG_2_, G_2_M) if a statistically significant portion of its regulatory targets belong to the *X *phase cell cycle-regulated genes defined by Spellman *et al*. [2] (see Methods). We found that a cell cycle TF may function in more than one cell cycle phase (see Table 1). On average, 86% (31/36) of our predictions have literature support. More specifically, 39% (14/36) of our predictions have experimental evidence and 47% (17/36) of our predictions are consistent with previous computational studies (see Table 1).

The following predictions have experimental evidence. Ace2 and Swi5 have been shown to control certain genes expressed in MG_1 _[16], supporting our prediction that Ace2 and Swi5 function in MG_1_. It is known that in the absence of Ndd1 and Fkh2, Mcm1 participates in the regulation of genes essential for cellular functions specific to late mitosis and early G1 [17,18], supporting our prediction that Mcm1 functions in MG_1_. Previous molecular and genetic analysis suggested that SBF (Swi4+Swi6) and MBF (Mbp1+Swi6) are activators of genes essential for cellular functions specific to late G_1 _[3,19], supporting our prediction that Mbp1, Swi4, and Swi6 function in G_1_. Two genomic studies [7,20] indicated the involvement of SBF in regulating S phase genes, supporting our prediction that Swi4 functions in S phase. Simon *et al*. [3] and Lee *et al*. [7] indicated the involvement of SBF and Fkh1/Fkh2 in regulating SG_2 _genes, supporting our predictions that Swi6, Fkh1 and Fkh2 function in SG_2_. Previous studies have demonstrated that Mcm1 interacts with Ndd1 and Fkh1/Fkh2 to regulate genes necessary for both entry into and exit from mitosis [21,22], supporting our prediction that Fkh1, Fkh2, Mcm1 and Ndd1 function in G_2_M.

### Identification of novel cell cycle-regulated genes

For each of the 17 identified cell cycle TFs, we look at their regulatory targets to find novel cell cycle-regulated genes. We regard a gene as a cell cycle-regulated gene if it is regulated by at least two of the 17 identified cell cycle TFs. The requirement for defining a cell cycle-regulated gene to be regulated by at least two rather than one cell cycle TF is to reduce the number of false positives. In total, we identified 178 novel cell cycle-regulated genes that are not in the set of 800 cell cycle-regulated genes identified by Spellman *et al*. [2]. We found that 64% (114/178) of the novel cell cycle-regulated genes have literature support. More specifically, 25% (45/178) of our predictions have experimental evidence and 39% (69/178) of our predictions are consistent with previous computational studies (see Additional file 1). Among the 178 identified novel cell cycle-regulated genes, 59 genes have no known function according to the *Saccharomyces *Genome Database [23]. We suggest that these 59 uncharacterized genes are involved in the cell cycle process. Two lines of evidence supported our predictions. First, 68% (40/59) of these genes have literature support. More specifically, 26% (15/59) of our predictions have experimental evidence and 42% (25/59) of our predictions are consistent with previous computational studies (see Additional file 1). Second, each of these 59 genes is regulated by at least two cell cycle TFs, and the TF-gene regulatory relationship is supported by at least three data sources: gene expression, TFBS, and ChIP-chip or/and mutant data. Let us consider three examples. According to the *Saccharomyces *Genome Database [23], *YJL160C *is a putative cell wall protein, *BUD7 *may be involved in the budding process, and *YCG1 *may be involved in mitotic chromosome condensation. However, the exact functions of *YJL160C*, *BUD7 *and *YCG1 *are still unknown [23]. Since cell wall synthesis, budding and chromosome condensation are all important to the cell cycle process [2], this suggests that *YJL160C*, *BUD7*, and *YCG1 *play a role in the cell cycle process, supporting our predictions.

## Discussion

### Advantages of our method

Our method has four features that make it more powerful than existing approaches. First, it can reduce false negatives in determining TF-promoter binding events from current ChIP-chip data. Most previous methods [7,8,24-27], except GRAM [25], used a stringent *p*-value threshold (≤ 0.001) to determine TF-promoter binding events in order to reduce the number of false positives, but it was at the expense of false negatives (~24%) [8]. In comparison, our method allows the *p*-value cutoff to be relaxed to 0.01 but requires that the promoter must have one or more binding sites of the TF. Therefore, using additional information provided by the TFBS data, our method can rescue some false negatives without substantially increasing the number of false positives. For example, we rescue 40 binding targets of Ace2. The promoter of each of these 40 genes has one or more binding sites of Ace2. However, their *p*-values of binding events in the ChIP-chip data are all larger than 0.001, so they would not have been identified using a stringent *p*-value of 0.001 (see Additional file 1 for the other 16 examples).

Second, it is known that ChIP-chip data can only indicate those TF-promoter binding events that happen in the same physiological condition in which the ChIP-chip experiments are conducted. Therefore, many plausible TF-promoter binding events may be missing in the current ChIP-chip data. In order to solve this problem, our method considers that a TF binds to a specific promoter if the disruption of the TF results in a significant change of the expression of the gene that has the specific promoter and if the promoter contains one or more binding sites of the TF. That is, using the information provided by the mutant and the TFBS data, our method can rescue many TF-promoter binding events that are missing in the current ChIP-chip data. For example, we rescue 16 binding targets of Ace2. The promoter of each of these 16 genes has one or more binding sites of Ace2 and the disruption of Ace2 results in a significant change of the expressions of all these 16 genes [10]. All these genes would not be identified as binding targets of Ace2 even when using a relaxed *p*-value of 0.01 in the ChIP-chip data (see Additional file 1 for the other 16 examples).

Third, our method can extract plausible TF-gene regulatory relationships from TF-promoter binding relationships. Most pervious methods [7,8,24-26] regard the TF-promoter binding relationships provided by ChIP-chip data as the TF-gene regulatory relationships. This may not be true because the binding of a TF to the promoter of a gene does not necessarily imply regulation. A TF may bind to the promoter of a gene but has no regulatory effect on that gene's expression. To solve this problem, our method uses a time-lagged dynamic system model of gene regulation to extract the TFs that have significant regulatory effects on the target gene's expression from all TFs that bind to the promoter of the target gene. Through this process, our method can extract plausible TF-gene regulatory relationships from TF-promoter binding relationships. Thus, in our method each TF-gene regulatory relationship is supported by at least three data sources: gene expression, TFBS, and ChIP-chip or/and mutant data. We found that, on average, 44% of the binding targets of the 17 identified cell cycle TFs are their regulatory targets (see Additional file 1). This ratio is slightly lower than Gao *et al*.'s estimation (58%) [27] and Wu *et al*.'s estimation (55%) [28], possibly due to our stringent requirement for a TF-gene regulatory relationship to be supported by at least three data sources, whereas in both previous studies the TF-gene regulatory relationship is only supported by two data sources: gene expression and ChIP-chip data.

**Figure 3:**
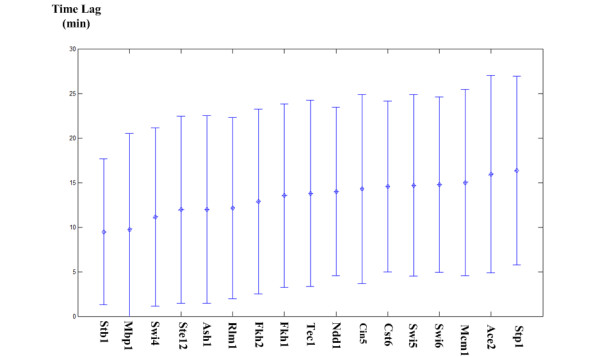
**The average time lag for a cell cycle to exert regulatory effects on its target genes**. The average and standard deviation of the time lag for each of the 17 identified cell cycle TFs to exert regulatory effects on its target genes are shown. For example, on average, it takes 9.5 minutes for Stb1 to exert regulatory effects on its target genes.

Fourth, our method can identify the time lag for a cell cycle TF to exert regulatory effects on its target genes. It is known that the regulatory effects of a TF on its target genes may have a time lag [29-33]. By using a time-lagged dynamical system model, our method takes the time lag into consideration, making it more realistic than those previous studies that did not allow a time lag [27,34-36]. As shown in Figure 3, the average time lag for each of the 17 cell cycle TFs to exert regulatory effects on its target genes was estimated.

### Performance comparison with existing methods

Four previous studies also tried to identify the yeast cell cycle TFs. Tsai *et al*. [12] identified 30 cell cycle TFs by applying a statistical method (ANOVA analysis) and Cheng *et al*. [14] identified 40 cell cycle TFs by applying another statistical method (Fisher's G test). Cokus *et al*. [37] identified 12 cell cycle TFs by applying linear regression analysis. Andersson *et al*. [38] identified 15 cell cycle TFs by applying rule-based modeling. Since these four approaches are different from ours, a performance comparison should be done. As suggested by de Lichtenberg *et al*. [15], we tested the ability of each of these five methods to retrieve the known cell cycle TFs annotated in the MIPS database [11]. Performance comparison was based on the Jaccard similarity score [39,40], which scores the overlaps between a method's output and the list of known cell cycle TFs (i.e., the true answers). Therefore, the higher the Jaccard similarity score, the better the ability of a method to retrieve the known cell cycle TFs. As shown in Table 2, our method has the highest Jaccard similarity score among the five methods. Therefore, our method outperforms the four existing methods.

**Table 2:** Performance comparison of five cell cycle TF identification methods to retrieve the known cell cycle TFs annotated in the MIPS database.

	TP	FP	FN	Jaccard similarity score
Our method	12	5	24	0.293

Tsai *et al*.'s method	13	17	23	0.245

Anderson *et al*.'s method	10	5	26	0.244

Cokus *et al*.'s method	9	3	27	0.231

Cheng *et al*.'s method	13	29	23	0.200

Performance comparison was based on the Jaccard similarity score [39,40], which scores the overlaps between a method's output and the list of known cell cycle TFs. Specifically, the Jaccard similarity score is defined as TP/(TP+FP+FN), where TP stands for true positives, FP for false positives, and FN for false negatives. Note that the higher the Jaccard similarity score, the better the ability of a method to retrieve the known cell cycle TFs.

### Robustness against different cell cycle gene expression datasets

**Figure 4:**
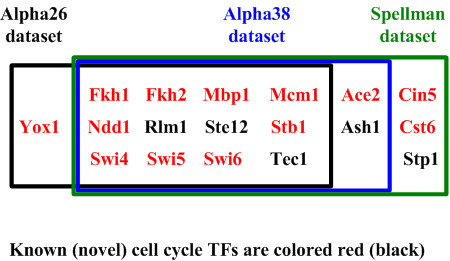
**The results of using different cell cycle gene expression datasets**. Our method identified 12, 14, and 17 cell cycle TFs using Pramila *et al*.'s alpha26 dataset, Pramila *et al*.'s alpha38 dataset, and Spellman *et al*.'s dataset, respectively. We found that among the 17 cell cycle TFs identified using Spellman *et al*.'s dataset, 14 TFs are also identified using Pramila *et al*.'s alpha38 dataset, and 12 TFs are also identified using Pramila *et al*.'s alpha26 dataset. This suggests that our method is robust against different cell cycle gene expression datasets.

We applied our method to two newer cell cycle gene expression datasets (alpha26 and alpha38) published by Pramila *et al*. in 2006 [41]. Both datasets are alpha-factor synchronized microarray time series spanning two cell cycles. The alpha26 dataset has a sampling interval of 10 minutes and a total of 13 data points, and the alpha38 dataset has a sampling interval of 5 minutes and a total of 25 data points. We found that among the 17 cell cycle TFs identified using Spellman *et al*.'s dataset, 14 TFs are also identified using the alpha38 dataset, and 12 TFs are also identified using the alpha26 dataset (see Figure 4). This suggests that our method is robust against different cell cycle gene expression datasets.

### Parameter settings of our method

The choices of both the relaxed *p*-value and time-lag parameter have biological meanings. Two previous papers [7,8] used a statistical error model to assign a *p*-value of the binding relationship of a TF-promoter pair. They found that if *p *= 0.001, the binding relationship of a TF-promoter pair is of high confidence and can usually be confirmed by promoter-specific PCR. If *p *> 0.01, the binding relationship of a TF-promoter pair is of low confidence and cannot be confirmed by promoter-specific PCR most of the time. However, if 0.001 <*p *≤ 0.01, the binding relationship of a TF-promoter pair is ambiguous and can be confirmed by promoter-specific PCR in some cases but not in the other cases. Our aim is to solve this ambiguity, so we choose 0.01 to be the relaxed *p*-value. We say that an ambiguous binding relationship of a TF-promoter pair is plausible if 0.001 <*p *< 0.01 and if the promoter contains one or more binding sites of the TF. As to the time-lag parameter, its value is chosen to make the maximal time lag approximately equal to two consecutive cell cycle phases because Simon *et al*. [3] found cases where a cell cycle TF that expresses in one phase of the cell cycle can regulate genes that function in the next phase.

We regard a gene as a cell cycle-regulated gene if it is regulated by at least two of the 17 identified cell cycle TFs. The requirement for defining a cell cycle-regulated gene to be regulated by at least two rather than one cell cycle TF is to reduce the number of false positves. When the stringent criterion is used, 64% (25% with experimental evidence and 39% with computational evidence) of the identified novel cell cycle-regulated genes are supported by the literature, whereas when the loose criterion is used, only 50% (8% with experimental evidence and 42% with computational evidence) of the identified novel cell cycle-regulated genes are supported by the literature. In this study, we want to be more conservative on calling a gene a "novel" cell cycle-regulated gene, so we aim to eliminate many false positives, though at the expense of some false negatives.

## Conclusion

We developed a method to identify cell cycle TFs in yeast by integrating current ChIP-chip, mutant, TFBS, and cell cycle gene expression data. We identified 17 cell cycle TFs, 12 of which are known cell cycle TFs. The remaining five TFs (Ash1, Rlm1, Ste12, Stp1, Tec1) are putative novel cell cycle TFs. Our predictions are supported by interactions (physical or genetic) data and previous studies. In addition, our method can assign each cell cycle TF to specific cell cycle phases in which the TF functions. We found that a cell cycle TF may function in more than one cell cycle phase. On average, 86% of our predictions have literature support (39% with experimental evidence and 47% with computational evidence). Besides, our method can identify the time lag for a cell cycle TF to exert regulatory effects on its target genes. By using a time-lagged dynamical system model, our method takes the time lag into consideration, which makes it more biologically realistic than those previous studies that did not allow a time lag. Moreover, we identified 178 novel cell cycle-regulated genes, 64% of which have literature support (25% with experimental evidence and 39% with computational evidence). Among the 178 novel cell cycle-regulated genes, 59 have no known function (i.e., they are uncharacterized). These 59 uncharacterized genes may now be annotated as cell cycle related genes, supported by the fact that 68% of our predictions have literature support (26% with experimental evidence and 42% with computational evidence). Furthermore, a high-confidence TF-gene regulatory matrix is derived as a byproduct of our method. Each TF-gene regulatory relationship in this matrix is supported by at least three data sources: gene expression, TFBS, and ChIP-chip or/and mutant data. Moreover, we compared the performance of our method with four existing methods and showed that our method has a better ability to retrieve the known cell cycle TFs. Finally, applying our method to different cell cycle gene expression datasets, we identify similar sets of TFs, suggesting that our method is robust.

## Methods

### Data sets and data preprocessing

We use four data sources in this study. First, the ChIP-chip data are from Harbison *et al*. [8]. They used genome-wide location analysis to determine the genomic occupancy of 203 TFs in rich media conditions. Second, the TFBS data are from MacIsaac *et al*. [9] and the YEASTRACT database [10]. MacIsaac *et al*. used evolutionarily conservative criteria to computationally identify the binding sites of many TFs in yeast. The YEASTRACT database includes a set of computational tools that can be used to identify complex motifs over-represented in the promoters of co-regulated genes. Third, the mutant data are from the YEASTRACT database [10]. The mutant data can tell us which gene's expression was changed significantly owing to the deletion (or mutation) of the gene that encodes a TF. The evidence may come from detailed gene by gene analysis or genome-wide expression analysis. Finally, the yeast cell cycle gene expression data are from Spellman *et al*. [2]. The alpha factor data set is used because it was shown to have a better data quality than the other data sets [15]. Samples for all genes in the yeast genome are collected at 18 time points (0, 7, 14, 21, ..., 119 minute), which cover two cell cycles. That is, each gene has a 18-timepoint gene expression profile. The cubic spline method [42] is then used to reconstruct the missing values and interpolate extra data points into the original time profile. Note that genes that have more than one missing value in their gene expression profiles are excluded in this study.

### Construction of a high-confidence TF-gene regulatory matrix

Using three data sources (ChIP-chip, mutant and TFBS data), we can construct a high-confidence TF-promoter binding matrix *B *= [*b*_*i*,*j*_], where *b*_*i*,*j *_= 1 if (1) the *p*-value for TF *j *to bind the promoter of gene *i *is ≤ 0.01 in the ChIP-chip data and the promoter of gene *i *contains one or more binding sites of TF *j *or (2) the disruption of TF *j *results in a significant change of the expression of gene *i *and the promoter of gene *i *contains one or more binding sites of TF *j*. Otherwise, *b*_*i*,*j *_= 0.

However, binding of a TF to the promoter of a gene does not necessarily imply regulation. Hence, additional information is required to solve this ambiguity inherent in the TF-promoter binding matrix. Using a time-lagged dynamic model of gene regulation, we can refine the TF-promoter binding matrix into a high-confidence TF-gene regulatory matrix. We consider the transcriptional regulatory mechanism of a target gene as a system with the regulatory profiles of several TFs as the inputs and the gene expression profile of the target gene as the output. The transcriptional regulation of a target gene is described by the following time-lagged dynamic system model [43-45]

(1) $$y[t]=\left(k+\sum_{i=1}^{N}d_{i}\cdot x_{i}[t-\tau_{i}]\right)-\lambda \cdot y[t-1]+\varepsilon [t]$$

where *y*[*t*] represents the target gene's expression profile at time point *t*, *k *represents the target gene's basal expression level induced by RNA polymerase II, *N *denotes the number of TFs that bind to the promoter of the target gene (inferred from the TF-promoter binding matrix *B*), *d*_*i *_indicates the regulatory ability of TF *i*, *x*_*i*_[*t*] represents the regulatory profile of TF *i *at time point *t*, *τ*_*i *_indicates the time lag for TF *i *to exert a regulatory effect on the target gene's expression, *λ *indicates the degrading effect of the target gene's expression value *y *[*t *- 1] at time point *t *- 1 on the target gene's expression value *y *[*t*] at time point *t *and *ε*[*t*] denotes the stochastic noise due to the modeling error and the measuring error of the target gene's expression profile. *ε*[*t*] is assumed to be a Gaussian noise with mean zero and unknown standard deviation *σ *The biological meaning of Equation (1) is that *y *[*t*] (the target gene's expression value at time point *t*) is determined by $k+\sum_{i=1}^{N}d_{i}\cdot x_{i}[t-\tau_{i}]$ (the production effect of RNA polymerase II and TF *i *at time point *t *- *τ*_*i*_, where *i *= 1,..., *N*) and -*λ*·*y*[*t *- 1] (the degradation effect of the target gene at time point *t *- 1).

It has been shown that TF binding usually affects gene expression in a nonlinear fashion: below some level it has no effect, while above a certain level the effect may become saturated. This type of binding behavior can be modeled using a sigmoid function. Therefore, *x*_*i*_[*t*] (the regulatory profile of TF *i *at time point *t*) is defined as a sigmoid function of *z*_*i*_[*t*] (the gene expression profile of TF *i *at time point *t*) [13,28,44,45]:

$$x_{i}[t]=f(z_{i}[t])=\frac{1}{1+\exp\left[-g\left(z_{i}[t]-A_{i}\right)\right]}$$

where *g *denotes the transition rate of the sigmoid function and *A*_*i *_denotes the mean of the gene expression profile of TF *i*. It is also known that the regulatory effect of a TF on its target genes may not be simultaneous but has a time lag [28-33]. Therefore, we incorporate a time lag term into our dynamic system model. The time lag *τ*_*i *_between TF *i *and the target gene *y *is determined by $\tau_{i}=\underset{q}{\arg\max}r(q)$, where *r*(*q*) is the correlation between $\vec{y}=\left(y[1],\cdots ,y[M]\right)$ (the expression profile of the target gene *y*) and ${\vec{x}}_{i}=\left(x_{i}[1],\cdots ,x_{i}[M]\right)$ (the regulatory profile of TF *i*) with a lag of *q *time points [28,29]:

$$\begin{array}{cc} r(q)=\left(\sum_{j=1}^{M-q}\left(y[j+q]-\bar{y}\right)\left(x_{i}[j]-{\bar{x}}_{i}\right)\right)/\left(\sqrt{\sum_{j=1}^{M-q}{\left(y[j+q]-\bar{y}\right)}^{2}}\cdot \sqrt{\sum_{j=1}^{M-q}{\left(x_{i}[j]-{\bar{x}}_{i}\right)}^{2}}\right), & q=0,1,...,Q \end{array}$$

where $\bar{y}=\left(\sum_{j=1}^{M-q}y[j+q]\right)/\left(M-q\right),{\bar{x}}_{i}=\left(\sum_{j=1}^{M-q}x_{i}[j]\right)/\left(M-q\right)$, *M *is the number of time points of the target gene's expression profile and *Q *is the maximal time lag of the TF's regulatory profile considered. The time lag may be interpreted as the time for a TF to exert a regulatory effect on its target gene's expression. The value of *Q *is chosen to make the maximal time lag approximately equal to two consecutive cell cycle phases because Simon *et al*. [3] found cases where a cell cycle TF that expresses in one phase of the cell cycle can regulate genes that function in the next phase.

After writing down the time-lagged dynamic system model of gene regulation, the next step is to estimate the unknown parameters in the model. We rewrite Equation (1) into the following regression form:

(2) $$y[t]=\left[\begin{array}{ccccc} x_{1}[t-\tau_{1}] & \cdots & x_{N}[t-\tau_{N}] & 1 & -y[t-1] \end{array}\right]\cdot \left[\begin{array}{c} d_{1}\\ \vdots \\ d_{N}\\ k\\ \lambda \end{array}\right]+\varepsilon [t]$$

Using the yeast cell cycle gene expression data from Spellman *et al*. [2], we can to get the values of {*x*_*i *_[*v*], *y *[*v*]} for *i *∈ {1, 2, ..., *N*}, *v *∈ {1, 2, ..., *M*}. Equation (2) at different time points can be put together as follows

(3) $$\left[\begin{array}{c} y[w]\\ y[w+1]\\ \vdots \\ y[M] \end{array}\right]=\left[\begin{array}{ccccc} x_{1}[w-\tau_{1}] & \cdots & x_{N}[w-\tau_{N}] & 1 & y[w-1]\\ x_{1}[w+1-\tau_{1}] & \cdots & x_{N}[w+1-\tau_{N}] & 1 & -y[w]\\ \vdots & \vdots & \vdots & \vdots & \vdots \\ x_{1}[M-\tau_{1}] & \cdots & x_{N}[M-\tau_{N}] & 1 & -y[M-1] \end{array}\right]\cdot \left[\begin{array}{c} d_{1}\\ \vdots \\ d_{N}\\ k\\ \lambda \end{array}\right]+\left[\begin{array}{c} \varepsilon [w]\\ \varepsilon [w+1]\\ \vdots \\ \varepsilon [M] \end{array}\right]$$

where $w=1+\underset{i=1,\ldots ,N}{\max}\tau_{i}$. For simplicity, we define the notations *Y*, Φ, *θ *and *e *to represent Equation (3) as follows

*Y *= Φ · *θ *+ *e*

where *Y *= [*y*[*w*] ... *y *[*M*]]^*T*^, Φ is the system matrix, *θ *= [*d*_1 _... *d*_*N *_*k λ*]^*T *^is the unknown parameter vector, and *e *is the error vector, The parameter vector *θ *can be estimated by the maximum likelihood (ML) method as follows [43]

$$\hat{\theta }={\left(\Phi^{T}\Phi \right)}^{-1}\Phi^{T}Y={\left[\begin{array}{ccccc} {\hat{d}}_{1} & \cdots & {\hat{d}}_{N} & \hat{k} & \hat{\lambda } \end{array}\right]}^{T}$$

Since *d*_*i *_stands for the regulatory ability of TF *i*, a large absolute value of *d*_*i *_means that TF *i *has a large effect on the target gene's expression. We consider TF *i *to be a true regulator of the target gene if its regulatory ability *d*_*i *_is statistically significantly different from zero. The test statistic $t=\frac{{\hat{d}}_{i}}{s\sqrt{u_{ii}}},$ a *t*-distribution with the degree of freedom equal to (*M *- *w *+ 1) - (*N *+ 2), is used to assign a *p*-value for rejecting the null hypothesis *H*_*0*_: *d*_*i *_= 0, where *u*_*ii *_is the *i *th diagonal element of the matrix (Φ^*T*^Φ)^-1 ^and $s=\sqrt{\frac{{\left(Y-\Phi \cdot \hat{\theta }\right)}^{T}(Y-\Phi \cdot \hat{\theta })}{\left(M-w+1)-(N+2\right)}}$ is an unbiased estimator of *σ *(the standard deviation of the stochastic noise *ε*[*t*]) [46]. The *p*-value computed by the *t*-distribution is then adjusted by the Bonferroni correction to represent the true alpha level in the multiple hypotheses testing [46]. Finally, TF *i *is said to be a true regulator of the target gene if the adjusted *p*-value *p*_*adjusted *_≤ 0.05.

From the above analysis, we can refine the TF-promoter binding matrix *B *= [*b*_*i*,*j*_] into a TF-gene regulatory matrix *C *= [*c*_*i*,*j*_]. In this matrix, *c*_*i*,*j *_= 1 if *b*_*i*,*j *_= 1 and if TF *j *is shown by the time-lagged dynamic system model to exert a significant regulatory effect on the expression of gene *i*. Otherwise, *c*_*i*,*j *_= 0.

### Identification of cell cycle TFs

From the high-confidence TF-gene regulatory matrix, the regulatory targets of each of the 203 TFs in yeast can be inferred. Then a TF is said to be a cell cycle TF if a statistically significant portion of its regulatory targets are in the set of 800 cell cycle-regulated genes identified by Spellman *et al*. [2]. The hypergeometric distribution is used to test the statistical significance [46,47]. The procedure for checking whether TF *j *is a cell cycle TF is as follows. Let *S *be the set of cell cycle-regulated genes identified by Spellman *et al*. [2], *G *be the set of genes that are regulated by TF *j *(inferred from the TF-gene regulatory matrix), *T *= *S *∩ *G *be the set of cell cycle-regulated genes that are also regulated by TF *j*, and *F *be the set of all genes in the yeast genome. Then the *p*-value for rejecting the null hypothesis (H_0_: TF *j *is not a cell cycle TF) is calculated as

(4) $$p=P(x\geq \left|T\right|)=\sum_{x\geq \left|T\right|}\frac{\left(\begin{array}{c} \left|S\right|\\ x \end{array}\right)\left(\begin{array}{c} \left|F\right|-\left|S\right|\\ \left|G\right|-x \end{array}\right)}{\left(\begin{array}{c} \left|F\right|\\ \left|G\right| \end{array}\right)},$$

where |*G*| means the number of genes in set *G*. This *p*-value is then adjusted by the Bonferroni correction to represent the true alpha level in the multiple hypotheses testing [46]. TF *j *is said to be a cell cycle TF if the adjusted *p*-value *p*_*adjusted *_≤ 0.05. This procedure is applied to each of the 203 TFs under study.

### Identification of the cell cycle phases in which a cell cycle TF functions

For each of the 17 identified cell cycle TFs, we want to determine in which cell cycle phases it functions. We regard that a cell cycle TF functions in the *X *phase (*X *= MG_1_, G_1_, S, SG_2_, G_2_M) if a statistically significant portion of its regulatory targets belong to the *X *phase cell cycle-regulated genes identified by Spellman *et al*. [2]. Equation (4) is again used to test the statistical significance. While *G *and *F *are defined as before, *S *now denotes the set of *X *phase cell cycle-regulated genes identified by Spellman *et al*. [2] and *T *= *S *∩ *G *now denotes the set of *X *phase cell cycle-regulated genes that are also regulated by the cell cycle TF under study. The *p*-value computed by Equation (4) is then adjusted by the Bonferroni correction to represent the true alpha level in the multiple hypotheses testing. We say that a cell cycle TF functions in the *X *phase (*X *= MG_1_, G_1_, S, SG_2_, G_2_M) if the adjusted *p*-value *p*_*adjusted *_≤ 0.05.

## Authors' contributions

WSW developed the algorithm, performed the simulation and wrote the manuscript. WHL conceived the research topic, provided essential guidance and revised the manuscript. All authors read and approved the final manuscript.

## Supplementary Material

Additional file 1 Supplementary Table 1 contains the detailed information of the 17 identified cell cycle TFs, the 178 novel cell cycle-regulated gene (including 59 uncharacterized genes), the results of using different cell cycle gene expression datasets, and performance comparison between our method and four existing methods. Click here for file
